# XRCC1 Polymorphisms and Urinary 8-Hydroxydeoxyguanine Levels Are Associated with Urothelial Carcinoma

**DOI:** 10.1371/journal.pone.0124066

**Published:** 2015-05-04

**Authors:** Chien-I Chiang, Ya-Li Huang, Chao-Yuan Huang, Horng-Sheng Shiue, Wei-Jen Chen, Yeong-Shiau Pu, Ying-Chin Lin, Yu-Mei Hsueh

**Affiliations:** 1 School of Public Health, College of Public Health and Nutrition, Taipei Medical University, Taipei, Taiwan; 2 Department of Public Health, School of Medicine, College of Medicine, Taipei Medical University, Taipei, Taiwan; 3 Department of Urology, National Taiwan University Hospital, College of Medicine National Taiwan University, Taipei, Taiwan; 4 Department of Chinese Medicine, Chang Gung Memorial Hospital and Chang Gung University College of Medicine, Taoyuan, Taiwan; 5 Department of Family Medicine, Shung Ho Hospital, Taipei Medical University, Taipei, Taiwan; 6 Department of Health Examination, Wan Fang Hospital, Taipei Medical University, Taipei, Taiwan; National Institute for Viral Disease Control and Prevention, CDC, China, CHINA

## Abstract

The aim of this study was to examine the associations between the combined effects of urinary 8-Hydroxydeoxyguanine (8-OHdG) level and polymorphisms of *XRCC1 Arg194Trp* and *XRCC1 Arg399Gln* on the risk of urothelial carcinoma (UC). We conducted a hospital-based case-control study that included 168 cases of UC and 336 age- and gender-matched healthy controls. We used polymerase chain reaction and restriction fragment length polymorphism analyses to examine the genotypes of *XRCC1* Arg194Trp and *XRCC1* Arg399Gln. We used a competitive *in vitro* enzyme-linked immunosorbent assay to determine urinary 8-OHdG levels. The *XRCC1* 399 Gln/Gln genotype and the *XRCC1 194* Arg/Arg genotype were positively correlated to UC (OR [95%CI] = 2.27 [1.20–4.27] and 1.59 [1.06–2.36], respectively). Urinary 8-OHdG levels were associated with UC in a dose-dependent manner. Participants with the *XRCC1* (Arg399Gln) Gln/Gln genotype or the G-C/A-C haplotype of *XRCC1* and a high urinary 8-OHdG level had a significantly higher risk of UC than those with the Arg/Arg + Arg/Gln genotype or the G-T haplotype and a low urinary 8-OHdG level. This is the first study to investigate the combined effect of urinary 8-OHdG level and *XRCC1* polymorphisms on UC risk. The findings are especially meaningful for participants with *XRCC1 399Gln* or *XRCC1 Arg194* genotypes and a high urinary 8-OHdG level, since these variables are associated with an increased risk of UC.

## Introduction

Worldwide, urothelial carcinoma (UC) is the most common malignancy of the genitourinary tract. UC originates from the urothelial epithelium and involves malignances of the renal pelvis, the ureter, the bladder and the urethra. In Taiwan, UC is the 12^th^ most frequently diagnosed type of cancer and the 14^th^ leading cause of cancer mortality. Men are diagnosed with new cases of UC more often than women by a ratio of 1.6:1 [1]. Cigarette smoking is the most important risk factor for UC and results in a 2- to 4-fold increased risk of UC [2,3]. The mechanism underlying cigarette smoking-induced UC remains unclear, but cigarette smoke contains more than 60 carcinogenic chemicals [4] that may induce carcinogenesis or increase proliferation of the bladder epithelium [5]. Our previous study showed that 4-(methylnitrosamino)-1-(3-pyridyl)-1-butanone, a nicotine-derived tobacco-specific nitrosamine, is linked to smoking-related UC [6]. Polycyclic aromatic hydrocarbons contained in cigarettes are also believed to be carcinogenic constituents that lead to the development of UC [7].

Many studies have shown that cigarette smoke can induce breaks in DNA strands and that reactive oxygen species (ROS) are the primary cause of these DNA lesions [8]. 8-hydroxydeoxyguanosine (8-OHdG), a hydroxyl product of deoxyguanosine that is generated by oxidative stress, is a biomarker that is used to assess oxidative DNA damage [9]. One study reported that cigarette smoke induced free radical damage in DNA and generated DNA base oxidation products, such as 8-OHdG, that are excreted in the urine [10]. The cigarette smoking-related oxidative DNA damage product 8-oxo-7,8-hydro-2’-deoxyguanosine resulted in mutagenic guanine base lesions through the action of ROS and ultimately lead to cell tumorigenesis [11]. Another recent study showed that 8-OHdG levels were correlated with environmental tobacco smoke exposure in a dose-dependent manner [12]. Also, when measured by the high-performance liquid chromatography-electrochemical detector method, 8-OHdG levels in DNA from leukocytes of bladder cancer patients were significantly higher than those in control patients [13]. Another study reported that urinary concentrations of 8-OHdG measured by competitive enzyme-linked immunosorbent assay (ELISA) were higher in patients with bladder cancer and patients with prostate cancer than in healthy control patients [14]. Our previous study also found that the urinary concentration of 8-OHdG in UC patients was significantly higher than that in control subjects [15].

X-ray repair cross-complementing group 1 (XRCC1) is an important DNA repair protein in the base excision repair (BER) pathway [16] and, for decades, it has been known to be associated with bladder cancer. Arg194Trp on exon 6 (rs1799782, C→T), Arg280His on exon 9 (rs25489, G→A), and Arg399Gln on exon 10 (rs25487, G→A) are the three most common single nucleotide polymorphisms (SNPs) in this gene and they have been extensively evaluated [17]. Recently, several meta-analyses reported significant associations between polymorphisms of *XRCC1* Arg194Trp [18,19], Arg280His [18,19], and Arg399Gln [18–20] and bladder cancer. However, to prove these associations, additional studies are needed that include improved design, different ethnic populations, and large sample sizes. Our recent study also found that *XRCC1* 399 Gln/Gln and 194 Arg/Arg DNA repair genes play an important role in poor arsenic methylation capacity, thereby increasing the risk of UC in non-obvious arsenic exposure areas [21].

Several studies have evaluated associations between polymorphisms of several DNA repair genes and the risk of bladder cancer. However, the correlation between urinary 8-OHdG combined with polymorphisms of DNA BER genes and UC remains unclear. In the present study, we evaluated whether polymorphisms of *XRCC1* (Arg399Gln) and *XRCC1* (Arg194Trp) modified the association between 8-OHdG levels and UC in areas of low arsenic exposure in Taiwan.

## Materials and Methods

### Study participants

We conducted a hospital-based case-control study using a study design that has been described previously [22]. Briefly, 168 UC cases and 336 age-matched and gender-matched healthy controls were recruited from the National Taiwan University Hospital and the Taipei Municipal Wan Fang Hospital from March 2007 to April 2009. All UC cases were diagnosed by histological confirmation, and none of the cases presented with other histology, such as squamous cell carcinoma, adenocarcinoma, sarcoma, lymphoma, or benign lesions. The healthy controls had no prior history of cancer. All study participants lived in Taiwan City, which is 200 to 300 km away from arsenic-contaminated areas. The participants drank tap water supplied by the Taipei Water Department of the Taipei City Government; the average arsenic concentration in tap water is 0.7 μg/L. The study was approved by the Research Ethics Committee of National Taiwan University Hospital and all participants provided informed consent before the questionnaire interview and biological specimen collection. This study complied with the World Medical Association Declaration of Helsinki.

### Questionnaire interview

All participants were interviewed by trained interviewers who used a structured questionnaire to collect personal information, including demographic and socioeconomic characteristics; lifestyle choices such as consumption of alcohol, tea, and coffee and cigarette smoking; and histories of family and personal diseases such as diabetes, stroke, and hypertension. Cigarette smoking history was classified as never, former, or current at the time of diagnosis. Ever-smokers included former and current smokers.

### Determination of urinary 8-OHdG levels

Spot urine samples were collected and placed in 50 mL acid-washed tubes. The samples were immediately transferred to a -20°C freezer until analysis. To ensure the stability of the samples, 8-OHdG levels were measured within 6 months of collection. Urinary specimens were centrifuged at 1,500 rpm for 10 min to remove particulates. The supernatants were used to measure 8-OHdG levels using a competitive *in vitro* ELISA kit (Japan Institute for the Control of Aging, Fukuroi, Japan) [23]. The detailed procedure was described previously [15]. The detection range of the ELISA assay was 0.5 to 200 ng/mL. The intra-assay coefficient of variance (CV) was 9.8% and the inter-assay CV was 6.7%. The urinary concentrations of 8-OHdG were corrected using individual urinary creatinine concentrations.

### Genotype determination

Genomic DNA was extracted using proteinase K digestion after phenol and chloroform extraction. Genotyping for *XRCC1* (Arg399Gln) and *XRCC1* (Arg194Trp) was determined by a polymerase chain reaction (PCR)-restriction fragment length polymorphism method [24]. Briefly, the following primers were used to amplify 615-bp and 491-bp PCR products for *XRCC1* (Arg399Gln) and *XRCC1* (Arg194Trp), respectively: 5’-TTGTGCTTTCTCTGTGTCCA-3’ (forward) and 5’-TCCTCCAGCCTTTTCTGATA-3’ (backward) for *XRCC1* (Arg399Gln) and 5’-GCCCCGTCCCAGGTA-3’ (forward) and 5’-AGCCCCAAGACCCTTTCACT-3’ (backward) for *XRCC1* (Arg194Trp). All PCR products were obtained in a total volume of 30 μL, which contained 40 ng DNA sample, 10X Taq buffer (Tris-HCl, PCR enhancers, (NH_4_)_2_SO_4_, and MgCl_2_), 2.5 mM dNTP, 2 μM of each primer, and 2 U Taq polymerase (Prime Taq, Genet Bio, Korea). The reaction sequence for *XRCC1* (Arg399Gln) included initial denaturation for 5 min at 94°C; 35 cycles of 94°C for 30 s, 59°C for 30 s, and 72°C for 30 s; and a final step of 72°C for 5 min. The reaction sequence for *XRCC1* (Arg194Trp) included initial denaturation for 5 min at 94°C; 35 cycles of 94°C for 30 s, 64°C for 30 s, and 72°C for 30 s; and a final step of 72°C for 5 min. PCR products were digested with *MspI* for *XRCC1* (Arg399Gln) (Arg/Arg: 375 and 240 bp; Arg/Gln: 615, 375, and 240 bp; Gln/Gln: 615 bp) and *PvuII* for *XRCC1* (Arg194Trp) (Arg/Arg: 491 bp; Arg/Trp: 491, 294, and 197 bp; Trp/Trp: 294 and 197 bp). The products were analyzed by electrophoresis on 2% agarose gels. For quality control, we randomly selected 5% of the samples and repeated the assay with a concordance of 100%. Different genotypes in each gene were confirmed by direct sequencing using an ABI PRISM model 3730 automated sequencer (Applied Biosystems, CA, USA).

### Statistical analysis

Continuous variables are presented as the mean ± standard error. We used the analysis of variance test to compare the urinary 8-OHdG levels among different *XRCC1* genotypes. The Hardy-Weinberg equilibrium (HWE) was assessed by a goodness-of-fit Chi-square test; this test examined possible genotyping errors for each SNP among the controls. The frequencies of *XRCC1* (Arg399Gln) and *XRCC1* (Arg194Trp) were fitted to HWE among the controls. Cutoff points for estimating dose-response relationships for continuous variables were the respective tertiles of the distributions in the controls. We performed a trend test by treating ordinal-score variables as continuous variables in the logistic regression model. We used multiple logistic regression models to estimate multivariate adjusted odds ratios (ORs) and 95% confidence intervals (CIs). The strength of the linkage disequilibrium (LD), shown by Lewontin *D’*, was calculated using the Haploview software package, version 4.1 [25]. We estimated *XRCC1* haplotypes using the expectation-maximization algorithm of the SAS/Genetics module. We used the additive model (synergy index) to evaluate the joint effects of urinary 8-OHdG level and *XRCC1* polymorphism or *XRCC1* haplotype on UC risk [26]. Thus, any two-sided *p*-value < 0.05 was considered statistically significant. When we calculated the UC risk of the *XRCC1 Arg194Trp* and *XRCC1 Arg399Gln* genotypes for multiple comparisons, a two-sided *p*-value < 0.025 was considered statistically significant. All analyses were conducted using the Statistical Analysis Software (SAS) statistical package (SAS, version 9.2, Cary, NC, USA).

## Results

Table 1 lists the sociodemographic characteristics and cigarette smoking statuses of all the study participants. Participants with a higher educational level had a significantly lower risk of UC than those with a lower educational level. Former smokers and ever-smokers had significantly higher risks of UC than non-smokers (OR [95%CI] = 3.32 [2.00–5.49] and 2.51 [1.59–3.95], respectively). The cases of UC were classified by disease stage: 62.5% were superficial, 25.0% were locally advanced, and 12.5% were metastatic; sixteen UC cases were not available for staging. The cases were also classified by tumor grade: 7.83% were grade I, 36.94% were grade II, and 45.22% were grade III/IV; eleven UC cases were not available for grading. Participants carrying the *XRCC1* (Arg399Gln) Gln/Gln genotype or the *XRCC1* (Arg194Trp) Arg/Arg genotype had a significantly higher risk of UC than those with the 399 Arg/Arg and Arg/Gln genotypes or with the 194 Arg/Trp + Trp/Trp genotype (OR [95%CI] = 2.27 [1.20–4.27] and 1.59 [1.06–2.36], respectively). The urinary 8-OHdG levels in UC and controls were 8.70 ± 1.45 and 5.10 ± 0.17, respectively (*p* = 0.01). Urinary 8-OHdG levels were significantly correlated with UC risk in a dose-dependent manner. The powers for age-gender adjusted models of *XRCC1* (Arg399Gln) (Arg / Arg + Arg / Gln vs. Gln / Gln), *XRCC1* (Arg194Trp) (Arg / Arg vs. Arg / Trp + Trp / Trp), and 8-OHdG level (≤ 3.45, 3.45–5.93, > 5.93) were 82.9%, 71.8%, and 85.8%, respectively. The powers for multivariate models of *XRCC1* (Arg399Gln) (Arg / Arg + Arg / Gln vs. Gln / Gln), *XRCC1* (Arg194Trp) (Arg / Arg vs. Arg / Trp + Trp / Trp), and 8-OHdG level (≤ 3.45, 3.45–5.93, > 5.93) were 71.9%, 64.1%, and 66.6%, respectively (data not shown).

**Table 1:** Sociodemographic characteristics, cigarette smoking status, cancer stage, tumor grade, XRCC1 genotypes, and urinary 8-OHdG level of urolethial carcinoma (UC) cases and healthy, non-UC controls.

Variables	UC cases (n = 168)	Controls (n = 336)	Age- and gender-adjusted OR (95% CI)	Multivariate OR^†^ (95% CI)
Gender				
Male	122 (72.62)	244 (72.62)	1.00	
Female	46 (27.38)	92 (27.38)	1.01 (0.66–1.55)	
Age (years)	62.05 ± 1.07	61.59 ± 0.76	1.00 (0.99–1.02)	
Educational level completed				
Illiterate/Elementary school	67 (39.88)	56 (16.67)	1.00 ^**§**^	
Junior/Senior high school	65 (38.69)	118 (35.12)	0.41 (0.26–0.67)**	
College and above	36 (21.43)	162 (48.21)	0.15 (0.09–0.26)**	
Cigarette smoking status				
Non-smoker	77 (45.83)	209 (62.20)	1.00 ^**§**^	
Former smoker	65 (38.69)	70 (20.83)	3.32 (2.00–5.49)**	
Current smoker	26 (15.48)	57 (16.96)	1.59 (0.88–2.86)	
Ever-smoker	91 (54.17)	127 (37.80)	2.51 (1.59–3.95)**	
Cancer stage^a^				
Superficial	95 (62.50)			
Locally advanced	38 (25.00)			
Metastatic	19 (12.50)			
Tumor grade^a^				
I	28 (17.83)			
II	58 (36.94)			
III/IV	71 (45.22)			
*XRCC1* (Arg399Gln) genotypes				
Arg/Arg	81 (48.21)	180 (53.57)	1.00 ^**§**^	1.00
Arg/Gln	61 (36.31)	132 (39.29)	1.03 (0.68–1.53)	0.89 (0.58–1.38)
Gln/Gln	26 (15.48)	24 (7.14)	2.44 (1.32–4.51)**	2.16 (1.12–4.18)**
Arg/Arg + Arg/Gln	142 (84.52)	312 (92.86)	1.00	1.00
Gln/Gln	26 (15.48)	24 (7.14)	2.41 (1.33–4.35)**	2.27 (1.20–4.27)^**b**^ ^,^ *
*XRCC1* (Arg194Trp) genotypes				
Arg/Arg	99 (58.93)	158 (47.02)	1.00 ^**§**^	1.00
Arg/Trp	55 (32.74)	145 (43.15)	0.60 (0.41–0.90)^**b**^ ^,^ *	0.61 (0.40–0.94)^**b**^ ^,^ *
Trp/Trp	14 (8.33)	33 (9.82)	0.68 (0.35–1.33)	0.71 (0.34–1.45)
Arg/Trp + Trp/Trp	69 (41.07)	178 (52.98)	1.00	1.00
Arg/Arg	99 (58.93)	158 (47.02)	1.62 (1.11–2.36)^**b**^ ^,^ *	1.59 (1.06–2.36)^**b**^ ^,^ *
8-OHdG level (ng/mg creatinine)	8.70 ± 1.45	5.10 ± 0.17	1.12 (1.06–1.18)**	1.11 (1.05–1.18)**
≤ 3.45	37 (22.02)	112 (33.33)	1.00 ^**§**^	1.00 ^**§**^
3.45–5.93	58 (34.52)	113 (33.63)	1.57 (0.96–2.56) ^**#**^	1.61 (0.96–2.70) ^**#**^
> 5.93	73 (43.45)	111 (33.04)	2.06 (1.26–3.38)**	1.85 (1.09–3.12)*

^**†**^Adjusted for age, gender, educational level, and cigarette smoking status.

^a^ Stage was unavailable for sixteen cases; grade was unavailable for eleven cases.

^b^ The UC risks of the *XRCC1 Arg194Trp* and *XRCC1 Arg399Gln* genotypes were adjusted for multiple comparisons; a two-sided *p*-value < 0.025 was considered statistically significant.

^**#**^0.05 ≤ *P* < 0.1;

*P < 0.05;

**P < 0.01;

^**§**^ *P* < 0.05 for trend test.

Table 2 provides the urinary 8-OHdG levels of all participants according to age, gender, and cigarette smoking status. Among both UC cases and controls, older participants (> 65 years old) had significantly higher urinary 8-OHdG levels than younger participants; males had significantly lower urinary 8-OHdG levels than females. Urinary 8-OHdG levels were not significantly different between ever-smokers and never-smokers.

**Table 2:** Comparison of urinary 8-OHdG levels with logarithmic transformed value between urothelial (UC) cases and healthy controls according to age, gender, and cigarette smoking status.

Variables	8-OHdG level (ng/mg creatinine)					
	Overall		UC cases		Controls	
	Mean ± SE	*p*-value	Mean ± SE	*p*-value	Mean ± SE	*p*-value
Age						
≤ 65 years	1.43 ± 0.04	<0.01	1.65 ± 0.08	0.09	1.32 ± 0.05	<0.01
> 65 years	1.68 ± 0.04		1.85 ± 0.08		1.59 ± 0.05	
Gender						
Male	1.49 ± 0.04	<0.01	1.06 ± 0.07	<0.01	1.43 ± 0.04	0.63
Female	1.67 ± 0.06		2.07 ± 0.07		1.47 ± 0.07	
Cigarette smoking status						
Non-smoker	1.55 ± 0.04	0.65	1.80 ± 0.07	0.30	1.46 ± 0.04	0.47
Ever-smoker	1.52 ± 0.05		1.68 ± 0.09		1.41 ± 0.06	

**Fig 1:**
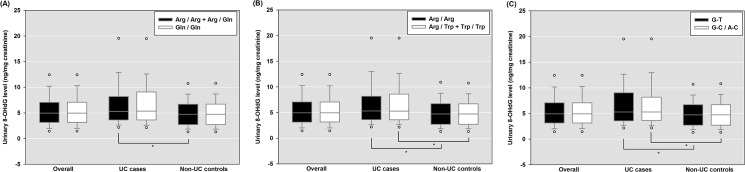
Comparison of urinary 8-OHdG levels between UC cases and healthy controls. (A) *XRCC1* (Arg399Gln) genotypes. (B) *XRCC1* (Arg194Trp) genotypes. (C) *XRCC1* haplotypes.

The LDs, assessed by Lewontin *D’*, of haplotypes of *XRCC1* (Arg399Gln) and *XRCC1* (Arg194Trp) polymorphisms were greater than 0.7, therefore, we tested the haplotype distributions of *XRCC1* in UC cases and controls using the Chi-square test (χ^2^ = 7.87, p = 0.05). The G-C haplotype of *XRCC1*, which contained the major alleles at the respective loci, occurred with the highest frequency in UC cases and controls (43.53% and 43.05%, respectively). Other haplotypes were seen less frequently in UC cases and controls: A-C (31.76% and 25.55%, respectively), G-T (22.84% and 30.17%, respectively), and A-T (1.87% and 1.23%, respectively). Haplotype A-T, which occurred at a frequency of less than 5%, was excluded from the haplotype analysis. Compared to the G-T haplotype of *XRCC1*, which was used as the reference group, the haplotypes of G-C and A-C were significantly associated with UC (OR [95%CI] = 1.41 [1.02–1.94], p < 0.05; data not shown). Fig 1 compares the urinary 8-OHdG levels between *XRCC1* (Arg399Gln) genotypes, *XRCC1* (Arg194Trp) genotypes, and *XRCC1* haplotypes. No significant differences in urinary 8-OHdG levels were observed among participants carrying different *XRCC1* genotypes or haplotypes.

Table 3 shows the associations between cigarette smoking status, 8-OHdG level, and UC risk stratified by *XRCC1* (Arg399Gln) genotypes, *XRCC1* (Arg194Trp) genotypes, and *XRCC1* haplotypes. Ever-smokers with the *XRCC1* (Arg399Gln) Arg/Arg+Arg/Gln genotype or the *XRCC1* (Arg194Trp) Arg/Trp+Trp/Trp genotype had a significantly higher risk of UC than non-smokers with the same genotypes. Ever-smokers with the *XRCC1* haplotype G-T or G-C/A-C also had a significantly higher risk of UC than non-smokers with the same haplotypes. Conversely, among participants with the *XRCC1* (Arg399Gln) Gln/Gln genotype, those with a high urinary 8-OHdG level (> 4.71 ng/mg creatinine) had a borderline significantly higher risk of UC than participants with a lower 8-OHdG level (≤ 4.71 ng/mg creatinine). Among participants with the G-C/A-C haplotype of *XRCC1*, a high urinary 8-OHdG level was significantly associated with UC risk (OR [95%CI] = 1.53 [1.08–2.15]).

**Table 3:** Associations between cigarette smoking status, 8-OHdG level, and urothelial carcinoma risk stratified by XRCC1 (Arg399Gln) genotypes, *XRCC1* (Arg194Trp) genotypes, and *XRCC1* haplotypes.

Variables	*XRCC1* (Arg399Gln) genotypes				*XRCC1* (Arg194Trp) genotypes				*XRCC1* haplotypes^¶^			
	Arg/Arg + Arg/Gln		Gln/Gln		Arg/Trp + Trp/Trp		Arg/Arg		G-T		G-C/A-C	
	Case / control	Multivariate OR (95% CI)	Case / control	Multivariate OR (95% CI)	Case / control	Multivariate OR (95% CI)	Case / control	Multivariate OR (95% CI)	Case / Control (%)	Multivariate OR (95% CI)	Case / Control (%)	Multivariate OR (95% CI)
8-OHdG level (ng/mg creatinine) ^‡^												
≤ 4.71	59 / 153	1.00	9 / 15	1.00	29 / 91	1.00	39 / 77	1.00	46.84 / 50.00	1.00	39.13 / 49.89	1.00
> 4.71	83 / 159	1.30 (0.84–2.01)	17 / 9	3.85 (0.98–15.13) ^#^	40 / 87	1.46 (0.78–2.71)	60 / 81	1.37 (0.78–2.43)	53.16 / 50.00	1.14 (0.64–2.03)	60.87 / 50.11	1.53 (1.08–2.15)*
Cigarette smoking status^†^												
Non-smoker	65 / 195	1.00	12 / 14	1.00	26 / 116	1.00	51 / 93	1.00	37.97 / 63.59	1.00	48.22 / 61.61	1.00
Ever-smoker	77 / 117	2.02 (1.21–3.37)**	14 / 10	0.87 (0.17–4.43)	43 / 62	3.23 (1.58–6.59)**	48 / 65	1.19 (0.60–2.36)	62.30 / 36.41	3.11 (1.60–6.05)**	51.78 / 38.39	1.60 (1.07–2.39)*

Haplotype frequencies of < 5% were excluded from the haplotype analysis.

^**¶**^XRCC1 haplotype: polymorphisms in two loci of the *XRCC1* are listed in the following order: Arg399Gln and Arg194Trp.

^†^Adjusted for age, gender, educational level, and 8-OHdG median level.

^‡^Adjusted for age, gender, educational level, and cigarette smoking status.

^**#**^0.05 ≤ *P* < 0.1;

*P < 0.05;

**P < 0.01.

Table 4 lists the joint effects of 8-OHdG level and *XRCC1* genotype or *XRCC1* haplotype on UC risk stratified by cigarette smoking status. For this analysis, the reference group contained participants with the *XRCC1* (Arg399Gln) Arg/Arg+Arg/Gln genotype and a low 8-OHdG level (≤ 4.71 ng/mg creatinine). The UC risk increased significantly in the following sequence: participants with the *XRCC1* (Arg399Gln) Arg/Arg+Arg/Gln genotype and a high urinary 8-OHdG level (> 4.71 ng/mg creatinine), participants with the *XRCC1* (Arg399Gln) Gln/Gln genotype and a low 8-OHdG level, and participants with the *XRCC1* (Arg399Gln) Gln/Gln genotype and a high urinary 8-OHdG level. The ORs (95%CIs) of UC in these participant groups were 1.31 (0.85–2.03), 1.49 (0.59–3.81), and 4.11 (1.75–11.08), respectively. The risk of UC was most pronounced in a dose-response relationship in participants with a high urinary 8-OHdG level and the *XRCC1* (Arg194Trp) Arg/Arg genotype or *XRCC1* haplotype G-C/A-C (*p* < 0.05 for the trend test). This phenomenon was also observed in participants who were non-smokers. However, none of the synergy indices of 8-OHdG level and *XRCC1* genotype or *XRCC1* haplotype on the risk of UC were statistically significant. The powers for the multivariate models of the effects of the interactions between 8-OHdG level (≤ 4.71 ng/mg creatinine vs. > 4.71 ng/mg creatinine) and the *XRCC1* (Arg399Gln) genotype, the *XRCC1* (Arg194Trp) genotype, and the *XRCC1* haplotype on UC risk in the entire participant group were 87.3%, 81.7%, and 57.7%, respectively (data not shown). Our study was adequately powered to test these models, with the exception of the combination of 8-OHdG level and the *XRCC1* haplotype

**Table 4:** The joint effects of 8-OHdG level and XRCC1 (Arg399Gln) genotypes, XRCC1 (Arg194Trp) genotypes, and XRCC1 haplotypes on urothelial carcinoma risk stratified by cigarette smoking status.

Variables		Overall		Cigarette smoking status			
				Non-smoker		Ever-smoker	
		Case / control	Multivariate OR^†^ (95% CI)	Case / control	Multivariate OR^‡^ (95% CI)	Case / control	Multivariate OR^‡^ (95% CI)
8-OHdG level (ng/mg creatinine)	*XRCC1* (Arg399Gln)						
≤ 4.71	Arg / Arg + Arg / Gln	59 / 153	1.00^§^	22 / 91	1.00^§^	37 / 62	1.00
> 4.71	Arg / Arg + Arg / Gln	83 / 159	1.31 (0.85–2.03)	43/ 104	1.44 (0.77–2.69)	40 / 55	1.22 (0.66–2.27)
≤ 4.71	Gln / Gln	9 / 15	1.49 (0.59–3.81)	3 / 9	1.48 (0.35–6.25)	6 / 6	1.48 (0.42–5.18)
> 4.71	Gln / Gln	17 / 9	4.41 (1.75–11.08)**	9 / 5	6.13 (1.73–21.72)**	8 / 4	2.71 (0.72–10.14)
8-OHdG level (ng/mg creatinine)	*XRCC1* (Arg194Trp)						
≤ 4.71	Arg / Trp + Trp / Trp	29 / 91	1.00^§^	9 / 60	1.00^§^	20 / 31	1.00
> 4.71	Arg / Trp + Trp / Trp	40 / 87	1.35 (0.74–2.44)	17 / 56	1.81 (0.72–4.53)	23 / 31	1.11 (0.48–2.57)
≤ 4.71	Arg / Arg	39 / 77	1.48 (0.81–2.71)	16 / 40	3.09 (1.21–7.91)*	23 / 37	0.84 (0.37–1.91)
> 4.71	Arg / Arg	60 / 81	2.24 (1.26–3.98)**	35 / 53	4.08 (1.72–9.72)**	25 / 28	1.24 (0.54–2.85)
8-OHdG level (ng/mg creatinine)	*XRCC1* haplotypes ^*¶*^	Case / control (%)		Case / control (%)		Case / control (%)	
≤ 4.71	G-T	11.14 / 15.44	1.00^§^	6.58 / 16.39	1.00^§^	15.00 / 13.89	1.00
> 4.71	G-T	12.65 / 15.44	1.03 (0.59–1.78)	13.16 / 15.18	1.91 (0.81–4.51)	12.22 / 15.87	0.63 (0.29–1.38)
≤ 4.71	G-C / A-C	29.82 / 34.48	1.11 (0.70–1.77)	26.32 / 31.33	2.56 (1.05–4.87)*	32.78 / 39.68	0.65 (0.34–1.22)
> 4.71	G-C / A-C	46.39 / 34.63	1.75 (1.11–2.76)*	53.95 / 37.11	3.28 (1.56–6.89)**	40.00 / 30.56	1.06 (0.56–2.00)

Haplotype frequencies of < 5% were excluded from the haplotype analysis.

^**¶**^Polymorphisms in two loci of the *XRCC1* are listed in the following order: Arg399Gln and Arg194Trp.

^†^Adjusted for age, gender, educational level, and cigarette smoking status.

^‡^Adjusted for age, gender, and educational level.

^**#**^0.05 ≤ *P* <0.1.

*P < 0.05.

**P < 0.01.

^**§**^ *P* < 0.05 for trend test.

## Discussion

To our knowledge, this is the first study to simultaneously evaluate the relationships between cigarette smoking status, urinary 8-OHdG levels, polymorphisms of *XRCC1* (Arg194Trp) and *XRCC1* (Arg399Gln), and UC risk. We showed that former smokers and ever-smokers had a 2- to 3-fold increased risk of UC compared to never-smokers. We also observed a significant dose-response relationship between urinary 8-OHdG and the risk of UC. In addition, we found that the *XRCC1* (Arg399Gln) Gln/Gln genotype and the *XRCC1* (Arg194Trp) Arg/Arg genotype were positively related to the risk of UC.

Cigarette smoking is associated with many disease states and is, therefore, an important issue worldwide. A recent prospective study showed that the hazard risk and 95%CI of UC was 2.00 (1.55–2.58) for former smokers and 3.81 (2.71–5.35) for current smokers [27]. This risk estimate was nearly identical to two previous cohort studies [3,28]. However, our present study found that the OR (95%CI) of UC was 3.32 (2.00–5.49) for former smokers and 1.59 (0.88–2.86) for current smokers. This difference may be due to patients’ disease statuses that inspired them to quit smoking cigarettes. Tobacco smoke contains a highly complex mixture of compounds that induce carcinogenesis through the formation of DNA adducts or oxidative DNA damage [29,30]. One previous study reported that significantly different DNA adduct levels were found in bladder cancer tissue between former smokers, current smokers, and never-smokers [31]. Further, DNA adduct levels in bladder tissue were significantly different between smokers and non-smokers [32]. Ghorbanihaghjo et al [29] reported that serum 8-OHdG levels of smokers were significantly higher than those of non-smokers. In contrast, another study reported that lymphocytes yielded lower 8-OHdG levels in smokers than in non-smokers [33]. However, in the present study, urinary 8-OHdG levels were not different between ever-smokers and never-smokers. These findings suggest that cigarette smoking may be related to UC risk through another mechanism, which needs further investigation.

8-OHdG is the most frequent base modification in DNA. One proposed mechanism for this modification is that H_2_O_2_ production generates oxidative attacks on telomere repeats (TTAAGGG); attacks on these repeats generate 8-OHdG and accelerate telomere shortening [34]. Expression of 8-OHdG reflects hydroxyl radical-mediated carcinogenesis, which plays a role in the development of bladder cancer. High levels of 8-OHdG in urinary bladder tumors affect the prognosis of patients [35]. Other studies have reported that 8-OHdG levels in DNA from leukocytes of bladder cancer patients were significantly higher than those from healthy controls [13] and that the levels of 8-OHdG in cancer tissues were significantly higher than in neighboring non-cancer tissues [36]. Our present study showed that increasing urinary 8-OHdG levels were related to an increasing risk of UC. However, the precise predictive value of 8-OHdG for the development of cancer remains unclear.

Much attention has been directed to genetic events in DNA that influence carcinogenesis, such as activation of oncogenes, inactivation of tumor suppressor genes, and defects of mismatch DNA repair genes. However, the process of carcinogenesis is still unclear. Seven meta-analyses discussed the association between polymorphisms of *XRCC1* Arg399Gln and bladder cancer in Asian populations and reported that the OR (95%CI) of bladder cancer for the Arg/Arg + Arg/Gln genotype versus the Gln/Gln genotype was 1.14 (0.88–1.48) [20]; for the Arg/Gln genotype versus the Gln/Gln + Arg/Arg genotype, 1.14 (0.98–1.33) [37]; for the Arg/Arg genotype versus the Gln/Gln + Gln/Arg genotype, 0.78 (0.43–1.41) for the entire Asian population and 0.68 (0.52–0.90) for smokers [19]; and the Gln/Gln genotype versus the Arg/Gln + Arg/Arg genotype, 0.65 (0.49–0.86) [38]. Three of the studies found no association between *XRCC1* Arg399Gln and bladder cancer [39–41]. However, in the present study, we found that participants who carried the *XRCC1* 399 Gln/Gln genotype had a significantly increased risk of UC compared to those with the Arg/Arg + Arg/Gln genotype (OR [95%CI] = 2.27 [1.20–4.27]). Previous studies showed that the *XRCC1* 399Gln allele may be associated with higher mutagen sensitivity, higher levels of carcinogen adducts [42,43], cell cycle delay [44], and mutation and sister chromatid exchanges [42]. Taken together, we conclude that the *XRCC1* 399 Gln/Gln genotype is related to an increased risk of UC.

We reviewed seven meta-analyses and found that subjects with the *XRCC1* Arg194Trp Arg/Trp + Trp/Trp genotype had a 1.2-fold (1.02–1.41) increased OR (95%CI) of bladder cancer compared to those with the Arg/Arg genotype [18,40]. The OR (95%CI) of bladder cancer for Asian people with the Trp/Trp genotype versus the Arg/Arg + Arg/Trp genotype was 1.97 (1.04–3.74) [37]; for the Trp/Trp + Arg/Trp genotype versus the Arg/Arg genotype, 1.33 (1.09–1.62) [19]; and for the Trp/Trp + Arg/Trp genotype versus the Arg/Arg genotype, 1.33 (1.03–1.72) [45]. Two studies found no association between *XRCC1* Arg194Trp and bladder cancer [38,39]. Here, we found that participants with the Arg/Arg genotype had a significantly higher risk of UC than those with the Arg/Trp + Trp/Trp genotype (OR = 1.59 [95%CI = 1.06–2.36]). Participants with the *XRCC1 194trp* allele had fewer bleomycinor benzo(a)pyrene diol epoxide-induced chromosomal breaks than those with the *XRCC1 Arg194 allele* [43]. *XRCC1 194trp* resides in the linker region that separates the DNA polymerase β domain from the poly (ADP-ribose) polymerase interaction domain [46]. It decreases the ability of DNA to repair itself and leads to increased accumulation of unpaired DNA damage that enhances apoptosis. This, in turn, reduces the probability that the cell cycle will replicate mutated DNA [47,48]. Thus, it is reasonable to hypothesize that *XRCC1 Arg194* is associated with an increased risk of UC. Little is known about how polymorphisms in the BER gene affect susceptibility to oxidative damage. The consequence of impaired DNA repair is the accumulation of various types of DNA lesions, including 8-OHdG. In this study, urinary 8-OHdG levels were not different among varied genotypes of *XRCC1* Arg399Gln and Arg194Trp and *XRCC1* haplotypes. However, participants with the *XRCC1* (Arg399Gln) Gln/Gln genotype or the G-C/A-C haplotype of *XRCC1* and a high urinary 8-OHdG level had a significantly higher risk of UC than those with the Arg/Arg + Arg/Gln genotype or the G-T haplotype and a low urinary 8-OHdG level. This suggests that the *XRCC1* (Arg399Gln) Gln/Gln genotype may be related to decreased effectiveness of BER through an alteration in the BRCA1 carboxyl-terminal domain of the XRCC1 [49], which reduces the binding of XRCC1 by poly (ADP-ribose) polymerase and leads to the accumulation of DNA damage. None of the synergy indices of the effect of high urinary 8-OHdG level and *XRCC1* (Arg399Gln) Gln/Gln genotype, *XRCC1* (Arg194Trp) Arg/Arg genotype, or *XRCC1* haplotype G-C/A-C on UC risk were statistically significant. The lack of adequate 8-OHdG repair may be associated with cancer [50], but this hypothesis needs further investigation.

There are some limitations of this study that need to be considered. First, this study only examined a small number of common polymorphisms involved in the BER pathway. Other polymorphisms or BER genes may actually be responsible for the observed responses. Some untyped SNPs may be in the LD with our selected SNPs. Due to the low frequency of the allele among the Han Chinese and the limited experimental methodology, we selected *XRCC1 Arg194Trp* and *XRCC1 Arg399Gln* as representative polymorphisms in *XRCC1*. Second, the design of this case-control study relied on an assumption of independence between genotype and urinary 8-OHdG and UC. We believe this assumption is appropriate because it is unlikely that participants knew their genotypes or modified their urinary 8-OHdG levels on the basis of this knowledge. However, we cannot exclude the possibility that the association between urinary 8-OHdG levels and UC in the present study is the result, and not the cause, of UC. Third, we did not collect complete information for cigarette smoking indices, such as the duration of cigarette smoking, the amount of cigarette smoking (packs/day), cumulative cigarette smoking (pack-years), and history of passive smoking. Additionally, clinical data, such as previous treatments and renal function, were not collected in this study. Last, the sample size was small, so statistical significance should be interpreted with caution.

In conclusion, to the best of our knowledge, this is the first study to investigate the combined effect of urinary 8-OHdG level and *XRCC1* polymorphisms on UC risk. The findings are especially meaningful for people with a high 8-OHdG level and the *XRCC1 399Gln* genotype or the *XRCC1 Arg194* genotype, which were associated with an increased risk of UC. Individuals may have inherently different UC risks based, in part, on genetic polymorphisms of BER and urinary 8-OHdG levels. These observations should be verified with additional studies that include larger sample sizes.
